# Pharmacological Treatment in Presbyopia

**DOI:** 10.3390/jcm11051385

**Published:** 2022-03-03

**Authors:** Andrzej Grzybowski, Varis Ruamviboonsuk

**Affiliations:** 1 Department of Ophthalmology, University of Warmia and Mazury, 10-720 Olsztyn, Poland; ae.grzybowski@gmail.com; 2 Institute for Research in Ophthalmology, Foundation for Ophthalmology Development, 61-553 Poznan, Poland; 3 Department of Biochemistry, Faculty of Medicine, Chulalongkorn University, Bangkok 10330, Thailand

**Keywords:** presbyopia, pharmacological, medical, correction, treatment

## Abstract

Pharmacological treatment of presbyopia may be an alternative for those who want a spectacle-free scenario and an easy-to-use method with lower risk of irreversible ocular adverse events. There are two main agents, miotics and lens softeners, investigated as agents for the pharmacological treatment. Miotic agents treat presbyopia by creating a pinhole effect which may increase the depth of focus at all working distances. The miotic agents have been studied for application to only one eye for monovision or both eyes. Their effect is temporary with common adverse events, such as headache and dim vision at nighttime, with no known long-term safety and efficacy. There have been studies on the miotic agents in combination with other agents for additive treatment effects or lessening adverse events, however, these combination effects are not clear. Lens softeners increase the elasticity of the lens, which is targeted at one of the etiologic mechanisms of presbyopia. There is only one lens softener being investigated in only a few trials. The results were inconclusive. The recent approval of 1.25% pilocarpine for treatment of presbyopia by the US FDA may be an important milestone for investigation of real-world data of pharmacological treatment of presbyopia.

## 1. Introduction

Presbyopia is a physiologically age-related reduction of accommodation leading to unsatisfied clarity of the near vision [1]. This condition usually starts after age of 45. In 2015, it was estimated that approximately 1.8 billion people were affected by presbyopia globally which was about 25% of the world population, and approximately 826 million people lacked proper visual correction. In the year 2030, the number of people with presbyopia is expected to be increasing to 2.1 billion people globally [2].

Accommodation is a mechanism enabling eyes to adjust their refraction power to focus on near objects. There are three main processes involved in accommodation. They are (1) ciliary muscle contraction, which in turn reduces the zonular tension and results in increased lens thickness, (2) pupillary constriction, and (3) convergence of both eyes [3,4]. The widely accepted cause of presbyopia is the stiffening of the lens, which limits lens thickening [5].

Presbyopia does not only affect the near vision, which is the distance between 20 and 40 cm from the eyes, but also affects the intermediate vision, which is the distance between 50 to 100 cm from the eyes [6].

Treatment and correction of presbyopia are still challenging since there are no drugs or procedures that can cause perfect vision at all distances without risk. Currently, there are several options to treat presbyopia: optical correction, including bifocal or progressive spectacles, monofocal or multifocal contact lenses, corneal or intraocular surgical procedures, and pharmacological treatment.

For optical correction with spectacles, such as monofocal, bifocal or multifocal lenses, they are common options because of easy access and non-invasive approach. However, spectacles are perceived by many patients as inconvenient [7,8,9]. Monovision contact lenses may deteriorate stereopsis since the lens is put on only one eye for near tasks. When there is a difference between focusing power of both eyes, the depth discrimination is affected [10]. Monovision associated with anisometropia of +2.00 diopters or higher may decrease stereoacuity [11]. Multifocal contact lenses may be an alternative to spectacles, however, they may cause discomfort, or inconvenience for some patients, particularly those who have never worn contact lens [12]. Contact lenses may also be related with a risk of serious ocular surface infections [13].

Surgical options, corneal or intraocular, are of increasing interest since they are based on most modern technologies. Corneal surgery, such as corneal monovision, corneal inlays, collagen shrinkage, or multifocal LASIK, was one of the common methods for presbyopia correction. They have shown successes in improving near vision, however, there are disadvantages, such as, reduction of intermediate or distance vision, decreased contrast sensitivity, dysphotopsia, or refractive regression. Due to these, some patients still need spectacles after the procedures [1,14]. Intraocular lenses (IOLs), such as monovision IOLs or multifocal IOLs, were also used for correction of presbyopia, with disadvantages, such as, dysphotopsia, or poorer intermediate vision, similar to corneal surgeries [14,15].

There are still risks of surgical complications, which are hardly reversible, and the best results are based on careful selection of patients. Moreover, good understanding of the limitation of the present technologies by patients and their trade-off nature are very important, for example, patients who gain spectacle-free of near or intermediate vision may experience some dysphotopsias in different lighting conditions or lose some sharpness of vision or stereopsis depending on the offered technology [16]. Finally, none of the present surgical technologies can offer full spectacles independence for the whole time of all activities.

Pharmacological treatment of presbyopia has been studied in recent years based on different drugs and different treatment regimens. Pharmacological treatment, in theory, may offer a benefit of having a spectacle-free condition with a lower risk of irreversible ocular complications, compared to surgery. In November 2021, U.S. FDA has approved 1.25% pilocarpine hydrochloride ophthalmic solution (AGN-190584) as an eye drop for treating presbyopia [17]. This is the first eye drop treatment of presbyopia that obtained U.S. FDA approval. It is possible that this approval may cause more interest in research on pharmacological treatment for presbyopia. On the other hand, there will be more data on efficacy and safety of the drug from the real-world experience, which may lead to better understanding of presbyopia. The aim of this article is to review current pharmacological treatment for presbyopia.

## 2. Methodology

We retrieved articles from PubMed database between January 2010 to November 2021 by using the following query terms: (presbyopia) AND (medical) AND ((treatment) OR (management) OR (therapy)), (presbyopia) AND (pharmacological) AND ((treatment) OR (management) OR (therapy)). We also searched for undergoing research listed in clinicaltrials.gov by using the following terms: Presbyopia | medication. We found 199 articles and 44 trials from these queries. We then excluded all articles that were not available in English and all studies on animals. After abstract screening, 30 studies focusing on pharmacological treatment of presbyopia were thoroughly reviewed.

## 3. Results

**Figure 1:**
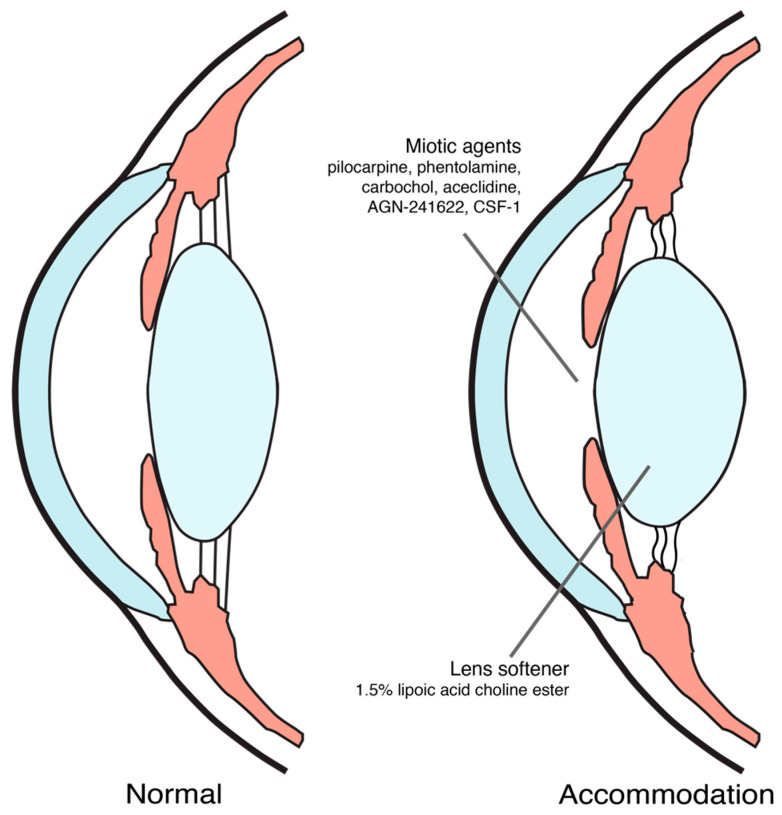
Pharmacological treatment for presbyopia mechanism of action.

Until now, proposed mechanisms of action for pharmacological treatment of presbyopia were inducing miosis and softening the lens [9,18,19], as shown in Figure 1. The ongoing trials on pharmacological treatment of presbyopia were summarized in Table 1.

### 3.1. Miotic Agents

Most of the current presbyopia pharmacological treatment options aim at inducing temporary miosis causing a pinhole effect to increase the depth of focus through parasympathetic pathway.

Miotic agents were used as either a monotherapy or in combination therapy with another miotic agent or other agents for treatment of presbyopia. Whereas most of them were used in combination therapy, the only two agents studied for monotherapy were pilocarpine and phentolamine.

#### 3.1.1. Pilocarpine HCl Ophthalmic Solution 1.25%—The U.S. FDA Approved Agent for Presbyopia

Pilocarpine is a miotic agent that has been used and studied in different concentrations, different forms and also as a combination with other drugs for presbyopia. It can induce miosis and ciliary body contraction which would also help in lens accommodation [31].

The daily use of pilocarpine hydrochloride ophthalmic solution 1.25% monotherapy in both eyes is the regimen which has been approved by the U.S. FDA. Pilocarpine ophthalmic solution was stored at a pH between 3.5 to 5.5 for its stability, the agent would have low bioavailability at this acidity. However, it was claimed that pilocarpine HCl ophthalmic solution 1.25% for the treatment of presbyopia could adapt to the pH of ocular surface within 1 min [32].

In phase 3 studies of this agent (GEMINI 1 and GEMINI 2), which were submitted for this approval, the eye drop was applied in each eye daily for 30 days and compared with placebo [33,34]. The key inclusion criteria of these studies were patients with presbyopia aged 40–55 years old, distance-corrected near visual acuity (DCNVA) between 20/40 to 20/100 and best distance correction between −4.00 to +1.00 D sphere and between −2.00 to +2.00 D cylinder. Participants were randomized into the treatment group (*n* = 375) and placebo group (*n* = 375). The mean age of participants was 49.6 ± 3.75 and 49.8 ± 3.38 in the treatment group and placebo group, respectively, and the majority of participants (85%) was Caucasian [35].

Since presbyopia affects both near and intermediate vision, outcome measures of both studies included four outcomes for near vision and one for intermediate vision. All of these outcomes were determined at different hours post-instillation on the 30th day of continuous use, therefore in the following sentences, ‘hours’ means hour post-instillation on the 30th day of continuous use. The outcomes for near vision were (1) the primary outcome, a percentage of participants with at least 3-line improvement in mesopic, high-contrast, binocular DCNVA without losing more than 5 letters of mesopic, high contrast, binocular corrected-distance visual acuity (CDVA) at 3 h, (2) a percentage of participants with at least 3-line improvement in mesopic, high-contrast, binocular DCNVA from 0.25 to 8 h, (3) changes from baseline in mesopic DCNVA from 0.25 to 10 h, and (4) a percentage of participants achieving photopic, binocular DCNVA of 20/40 or better at 1 h and 3 h. The outcome for intermediate vision was changes from baseline in photopic, high-contrast, binocular distance-corrected intermediate visual acuity (DCIVA) at 1 h and 3 h [33,34,35].

The pooled results from both studies showed that, for the primary outcome, the percentage of participants in the treatment group who gained at least 3-line in mesopic, high-contrast, binocular DCNVA was significantly greater than the percentage of those in the placebo group (28.27 vs. 9.6, *p* < 0.01). Moreover, the percentage of participants who received this agent and gained at least 3-line of DCNVA was significantly greater than that of participants who received placebo at 0.25 h (16.80 vs. 8.00, *p* ≤ 0.0018), 0.5 h (33.33 vs. 9.67, *p* < 0.0001), 1 h (39.20 vs. 13.87, *p* < 0.0001), 3 h (29.07 vs. 9.60, *p* < 0.0001), and 6 h (17.33 vs. 9.33, *p* ≤ 0.0018) [33,34,35].

In terms of the mean change from baseline of mesopic DCNVA, participants treated with 1.25% pilocarpine had more DCNVA letter gained than those treated with placebo from 0.25 to 10 h. The percentage of participants achieving photopic, binocular DCNVA of 20/40 or better in the treatment group was also greater than those in the placebo group [33,34,35]. Additionally, from the GEMINI 1 results, about 75% of participants in the treatment arm gained at least 2-line improvement in mesopic DCNVA [36].

For the outcome of intermediate vision, the patients in the treatment group significantly improved their photopic, high-contrast, binocular DCIVA at 1 h and 3 h [33,34,35].

The most common adverse event related to this agent in the pooled data was headache at 14.9%. Conjunctival hyperemia, vision blur and eye pain were reported in 5.1%, 4.5% and 4.3% of patients in the treatment group, respectively. There were no other serious adverse events in the treatment group [33,34,35].

Due to the induced miosis might affect night vision, a phase 3 study on the impact of 1.25% pilocarpine hydrochloride ophthalmic solution on night-driving performance has also been conducted [21].

#### 3.1.2. Other Miotic Agents

Another form of pilocarpine topical drug as ophthalmic cream monotherapy for presbyopia has also been studied. There is an ongoing phase 2 study on various concentrations of this pilocarpine ophthalmic topical cream on their safety and efficacy after using the medication twice a day for 28 days and assessing DCNVA as the primary outcome [28].

A phase 3 study on a 2% pilocarpine ophthalmic solution spray, another alternative mode of delivery, has been conducted with the primary outcome measures as DCNVA at 120 min after instillation [29].

Phentolamine is a nonselective alpha-adrenergic antagonist that has been studied for monotherapy. An ophthalmic solution of 1% phentolamine mesylate could decrease pupil diameter and create pinhole effect. The results from a phase 2b study by Pepose et al., using this drug once daily in the evening for 14 days, compared with placebo, showed that patients in the treatment group had at least 1-line improvement in DCNVA at day 15. There was no difference in conjunctival hyperemia between both treatment and placebo groups [37].

For the combination of a miotic agent with another miotic agent or other agents, the aim is either to gain addition effect between each drug or to reduce the side effects from the miotic agent.

There are several studies evaluating pilocarpine in combination with anti-inflammatory agents or vasoconstrictive agents to minimize adverse events of conjunctival injection or ocular inflammation that may be induced by pilocarpine. An ongoing randomized controlled study has been initiated to compare between monotherapy of miotic agents and the combination of two miotic agents. There were four treatment groups for the comparison in this study: phentolamine and pilocarpine, phentolamine alone, pilocarpine alone and placebo, DCNVA in these treatment groups were compared at baseline and at 6 h [25].

A combined eye drop between pilocarpine and diclofenac (Benozzi’s Method) had been patented and studied by Benozzi et al. [38]. In their retrospective case series, this combined drug was used twice daily as presbyopia treatment. At 1 year, the average of uncorrected distance visual acuity (UNVA) of 96 patients was improved from 4.42 at baseline to 1.18 in Jaeger units. There was still an improvement in UNVA after 8 years of follow-up of 144 patients, from 4.72 at baseline to 1.36. In the follow-up period of 8 years, an average of 70% of patients receiving the eye drop had an improvement in UNVA. Approximately 26% of patients reported dimness after instillation, which was resolved after 12 months, whereas 12.9% reported headache [39]. In another retrospective case series by Benozzi et al., which included patients who received this combined eye drop for presbyopia and were followed-up for at least 2 years, all patients improved their UNVA, while there was no difference in uncorrected distance visual acuity (UDVA) compared with baseline [40].

A combination of pilocarpine, phenylephrine, polyethyleneglycol, nepafenac, pheniramine and naphazoline into a single eye drop had also been studied. In a study by Vargas et al., the results showed that the participants’ UNVA at 2 h after instillation was statistically improved from the baseline. However, 14 out of 117 participants reported headache and one participant could not tolerate this side effect [41].

Another combination of five drugs into a single eye drop has also been studied. A phase 1 study on pilocarpine, brimonidine, oxymetazoline, hyaluronic acid and bromfenac (PBOHB) compound has been initiated. This study assessed UNVA improvement at 1 h after binocular instillation of the PBOHB compound, and assessed adverse reactions of this compound [27].

An alpha 2 adrenergic receptor agonist, AGN-241622, which prevents pupil dilation, has been investigated in a phase 1/2 study to evaluate its pharmacokinetics, safety, and efficacy by using the drop as either unilateral or bilateral. These regimens were compared with 1.25% pilocarpine ophthalmic solution, and a placebo on both healthy participants and participants with presbyopia [22].

In a study by Abdelkader, this researcher used 2.25% carbachol, a parasympathomimetic agent, followed by 0.2% brimonidine, an alpha agonist, to induce miosis in the patient’s nondominant eye and compare its efficacy with placebo on near vision improvement. From a total of 48 eyes, near visual acuity (NVA) of patients in the treatment group was significantly improved from baseline, however, the best mean change in NVA was at one hour after instillation and the NVA gradually declined. This study is one of few studies in which the agent to treat presbyopia is applied to only one eye to minimize bilateral dim vision. Despite of this monocular therapy, one out of thirty patients in the treatment group reported difficulty in low luminosity scenario [42].

Another study by Abdelkader and Kaufman compared between using a combination of 3% carbachol and 0.2% brimonidine in a single drop versus using 3% carbachol followed by 0.2% brimonidine after 5 min or 3% carbachol alone or 0.2% brimonidine alone in the same patients with a week of washout period between each regimen. The comparison in terms of NVA was measured at hour 1, hour 2, hour 4, and hour 8 after instillations. The combined carbachol and brimonidine solution significantly improved NVA from baseline than other regimens at each measurement. The carbachol alone regimen also significantly improved NVA compared with baseline, however, the brimonidine alone regimen did not improved NVA at any time point compared with baseline [43]. These results might show that it was the effect of carbachol which was essential to improve NVA.

Another phase 2 study on a combined drug of carbachol and brimonidine (Brimochol), Brimochol F and carbachol monotherapy was also conducted [44]. There was no availability of concentration of both medications in this study. Both Brimochol and Brimochol F improved at least 12 letters of NVA at 9 h after instillation. In addition, there were some minor adverse events, such as burning sensation, headache, brow ache, which were well tolerated by patients [45]. A phase 3 study on Brimochol in patients with emmetropic phakic and pseudophakic presbyopia has been started. This study aimed at recruiting 450 patients who are 45 to 80 years old [26].

CSF-1 has been investigated in a phase 2a randomized placebo-controlled crossover study using the agent in both eyes once daily for two weeks. The results showed the rate of two-line improvement in UNVA was significantly higher with CSF-1 (41.7% versus 22.2% in high illumination, and 41.7% versus 16.7% in low illumination). No moderate or severe adverse events were reported [7]. Currently, two phase 3 studies, comparing CSF-1 and placebo on best distance-corrected visual acuity (BDCVA) improvement, are undergoing [46].

Aceclidine, another miotic agent, was combined with tropicamide, a commonly used pupillary dilating drop, in a phase 2 randomized controlled study on its efficacy and safety. It was shown that binocular use of either the combined eye drop of aceclidine and tropicamide or aceclidine alone improved NVA at 1-h post-treatment, while no improvement was found in the placebo group. In addition, in comparison between this combined drug and aceclidine alone, there was no statistical difference in terms of both efficacy and adverse events between these two treatment regimens [47].

### 3.2. Lens Softeners

Loss of lens elasticity is associated with advanced age and presbyopia. This may be related to an increase in disulfide bonds formation in the collagen of aging lens, possibly due to oxidative stress [48]. Inducing miosis might be an effective mechanism for treatment of presbyopia by creating a pinhole effect but it might not address the etiology of this condition. Lens softening, on the other hand, might be an agent that could make a change at its pathophysiology.

There was in vitro evidence showing that lipoic acid, an antioxidant, could reduce disulfide bonds in the lens protein, and thus increase lens elasticity [48]. However, lipoic acid alone as an eye drop had limited ocular penetration because of its lipid solubility [49]. Bonding lipoic acid with choline allows the drug to better penetrate into the aqueous humor [50].

In a study by Korenfeld et al., 1.5% lipoic acid choline ester ophthalmic solution (UNR844) was compared with placebo for DCNVA and adverse events. The inclusion criteria of this study were patients with presbyopia aged 45–55 years, monocular DCNVA worse than 20/40 in each eye, best-corrected distance visual acuity of 20/20 or better, and manifested spherical equivalent between −4.00 to +4.00 D. From a total of 75 patients enrolled, they were randomized into the treatment group and the placebo group with 2:1 ratio, resulting in 50 patients in the former and 25 patients in the latter. The mean age of the treatment group and the placebo group was 50.1 ± 3.2 and 51.4 ± 3.0, respectively; 70.67% of patients were Caucasians [50].

The treatment group were given 1.5% lipoic acid choline ester ophthalmic solution unilaterally in their nondominant eye twice daily on day 1–7, and then given bilaterally twice daily on day 8–91. There was significantly improved in DCNVA from baseline in the treatment group compared with the placebo group over the course of 91 days (0.198 vs. 0.099 LogMAR VA units). Moreover, about a third of the patients with 1.5% lipoic acid had a sustained bilateral improvement of at least 1-line DCNVA at day 301. In terms of adverse events, the most common was eye irritation at 6%. Patients also reported asthenopia, eye pruritus, and foreign body sensation. For ocular safety, there were no clinical changes in non-dilated pupil diameter, ocular comfort, intraocular pressure (IOP), distance vision, or other ocular findings in the treated eyes [50].

There was another phase 2 multi-centered, double-masked, placebo-controlled, randomized, parallel-group study on 1.5% lipoic acid choline ester chloride compared with placebo. The mean age of the participants was 53.9 ± 4.97 and 54.2 ± 5.01 years old in the treatment group and the placebo group, respectively, while 81.5% of participants were Caucasians. The results showed that the changes in binocular DNCVA from baseline of the treatment group and placebo group were not statistically significant (6.1 vs. 4.5 letter gained, *p* = 0.1832) [51]. There were no differences in systemic and ocular adverse events between both groups.

A multi-centered, double-masked, placebo-controlled, randomized, parallel-group phase 2b study on administrating UNR844 at different doses twice daily for three months has already been initiated. DCNVA after three months was assigned as the primary outcome measure. The follow-up period was 9 months after drug cessation [30].

## 4. Discussion

The overview of treatment options for presbyopia is shown in Table 2. Since there is still no standard treatment of presbyopia, pharmacological treatment may be an alternative to those who do not want to wear spectacles, contact lenses, or have surgical procedures. However, disadvantages of pharmacological treatment are risk of ophthalmic and systemic adverse events, such as dim vision, poor night vision, conjunctival hyperemia, and headache. Long-term application is required if patients would want to avoid wearing spectacles completely.

There is some evidence suggesting pharmacological treatment is able to improve NVA of patients with presbyopia although all the data were from clinical trials with limited sample size and follow-up period [36,37,41,43,50]. All of these studies compared pharmacological treatment with placebo, not compared with spectacles correction. Studies on pharmacological treatment of presbyopia are in an early stage with majority of them were conducted within the last 5 years. Many studies are ongoing with results expected within a few years. In addition, although only two main groups of pharmacological agents have been investigated, more studies on these agents and new agents are expected in the future.

### 4.1. Miotic Agents

Although most studies on miotic agents were prospective studies, they neither had a large sample size, nor a long follow-up period. Only the GEMINI 1 and GEMINI 2 study of 1.25% pilocarpine had more than 100 participants in both treatment and placebo arms, however, this study had a study period of 30 days, therefore the long-term effect of this agent is not known. Although the pooled data of both studies showed that participants treated with pilocarpine HCl ophthalmic solution 1.25% improved in both near and intermediate vision, the percentage had not reached 50% yet [33,34,35]. There were only retrospective data on long-term use of 8 years of a miotic agent, pilocarpine combined with diclofenac, in approximately 150 patients [39,40]. However, there is no detail on the concentration of each of the agents in these retrospective studies.

Most of the studies conducted in wide range of patients with presbyopia. Since presbyopia is a progressive condition in which power of accommodation is gradually lost in older patients, studies on different specific age groups, for example, patients with presbyopia whose age are between 45 and 50 years old, or more than 60 years old, might be helpful to understand the treatment efficacy in these different age groups [41].

There is still no standard on applying miotic agents as unilateral or bilateral treatment. The unilateral application requires familiarity of monovision from patients. There was still no direct comparison between the efficacy of unilateral or bilateral instillation of miotic agents. Application of miotic agents for presbyopia may also have a limitation in those aging patients who have cataract since miosis may worsening their vision.

It should be emphasized that pilocarpine which was U.S. FDA approved for treatment of presbyopia has a concentration of 1.25% which is different from pilocarpine for treatment of glaucoma which has a concentration of 2% or 4%.

There was not enough data to conclude that other miotic agents, such carbachol or brimonidine, can be given to treat presbyopia with similar efficacy and safety, compared with pilocarpine. In addition, there was still inconclusive evidence that compounds for presbyopia treatment which were made to minimize adverse effects in counteract with, or to provide additional pinhole effect to, a miotic agent were beneficial [38].

Patients receiving miotics should be cautious in low light conditions because the small pupil might not allow more light entering the eyes in these conditions [9,31,42]. Additionally, retinal detachment is a possible serious adverse event associated with any miotics, although there have not yet been studies found direct causal relationship between miotics and retinal detachment [52]. Patients, particularly those who are at risk of retinal detachment, such as myopes, pseudophakic, and elderly patients, should be informed before using them [53].

Inducing miosis may not be an ideal solution for treatment of presbyopia since miosis itself is not a physiologic condition of the eyes. After the approval of 1.25% pilocarpine ophthalmic solution in the US, users had mixed experiences on the drug. According to initial data from online websites, some users reported that the drug improved their near vision for about two hours while some users did not encounter the difference from before instillation [54]. There have not been any data from other countries since it was approved only in the US. Moreover, for the real-world user experiences to be reliable and citable, they should be collected systematically, reviewed, and reported in academic journals.

### 4.2. Lens Softeners

Until now to the best of our knowledge, there is only UNR844, an anti-oxidant agent, which was used as lens softener for treatment of presbyopia. There have been only two completed studies and one ongoing clinical trial on UNR844 [30,50,51]. The results from the two completed studies are contradicted with each other. Although the first study showed favorable results, the second study, which was unpublished and reported on clinicaltrials.gov, showed contradictory evidence.

In terms of visual acuity measured as DCNVA, the first study which was by Korenfeld et al. showed that the treatment group gained approximately 5 letters [50], while the second unpublished study showed only 1.6-letter-letter gained [51]. The sample size in these studies were similar with approximately 70 subjects [50,51]. Still, this size was relatively small, and the results might not be compared directly. Future studies with a larger sample size would be beneficial for judging the efficacy of this agent. Another interesting detail from both studies was the difference in the age of enrolled patients. Patients in the first study were generally younger than those in the second study, especially in the treatment group (approximately 50 vs. 54) [50,51]. Thus, it is possible that younger patients might respond to UNR844 better than older patients; additional studies are required to test this hypothesis.

Lens softeners should theoretically be a better treatment option than miotics because they addressed the etiology of presbyopia and did not alter the normal physiological function of the eyes. Unfortunately, there have been only a few studies on this agent, more new agents addressing other areas related to pathophysiology of presbyopia should be encouraged.

## 5. Conclusions

With the recent U.S. FDA approval of 1.25% pilocarpine for treatment of presbyopia, this agent is becoming an alternative option for those in the US who may not want to wear spectacles for near tasks. It is possible that this agent may be approved in other countries and more patients may be able to access this agent in the future. This would give an opportunity to investigate on safety and efficacy of this agent in the real world, particularly for long-term use, which is essential.

There are ongoing studies on other miotic agents, as monotherapy or in combination with other agents as treatment of presbyopia. The results of these studies should be available soon. Some of the already published studies showed inconclusive evidence on benefits of these compounds. Lens softener, another pharmacological agent under investigation for presbyopia, addresses this condition at its etiology. The initial result of the agent was encouraging; however, more studies, especially a phase 3 study, are required for assessing its safety and efficacy.

Pharmacological treatment of presbyopia is, without a doubt, one of the promising fields in research in ophthalmology since all people who are more than 45 years old will have this condition eventually.

## Figures and Tables

**Table 1:** Ongoing studies on pharmacological treatment for presbyopia from clinicaltrials.gov (at the time of writing).

Drugs	N	Study Design	Instillation Method	Primary Outcome	NCT ID	Phase
	Miotic agents					
1.25% pilocarpine [20]	230	Multi-center, double-masked, randomized, vehicle-controlled, parallel-group study	Twice daily binocularly for 14 days	Percentage of participants gaining 3 lines or more in mesopic, high contrast, binocular DCNVA at day 14	NCT04983589	3
1.25% pilocarpine [21]	54	Randomized, double-masked, crossover study	Twice daily binocularly for 14 days	Overall Composite Driving Z score approximately 1 h after study intervention instillation	NCT04837482	3
AGN-241622 [22]	144	Phase 1/2, dose-ascending, multi-center, randomized, double-masked, vehicle-controlled study	Single drop binocularly	Number of patients experiencing a treatment emergent adverse event after single administration of AGN-241622 at day 2 and day 14	NCT04403763	1/2
CSF-1 [23]	300	4-visit, multi-center, randomized, double-masked, vehicle-controlled study	Twice daily binocularly for 2 weeks	Percentage of subjects with a ≥ 3-line gain in BDCVA at 40 cm and no loss in BDCVA ≥ 5 letters at 4 m at day 8	NCT04599933	3
CSF-1 [24]	300	4-visit, multi-center, randomized, double-masked, vehicle-controlled study	Twice daily binocularly for 2 weeks	Percentage of subjects with a ≥ 3-line gain in BDCVA at 40 cm and no loss in BDCVA ≥ 5 letters at 4 m at day 8	NCT04599972	3
1% phentolamine [25]	150	Randomized, quadruple-masked, parallel-group study	Single drop binocularly	Percentage of subjects with ≥15 letters of improvement in photopic binocular DCNVA after 6 h	NCT04675151	2
Carbachol and brimonidine [26]	450	Multi-center, randomized, double-masked study	Single drop binocularly	Percentage of subjects with 3-line gains in near VA with the loss of at least 1 line in DVA	NCT05135286	3
PBOHB compound [27]	11	Single group study	Single drop binocularly	Jaeger near uncorrected visual acuity improvement after 1 h	NCT05006911	1
Pilocarpine cream [28]	120	Multi-center, randomized, double-masked, placebo-controlled, parallel group study	Once daily binocularly for 28 days	Binocular DCNVA after 28 days	NCT05124275	2
Pilocarpine Spray [29]	139	Randomized, triple-masked, crossover, placebo-controlled study	Single drop binocularly	Proportion of subjects gaining ≥ 15 letters in mesopic, high contrast, binocular DCNVA at 120 min post-treatment	NCT05114486	3
	Lens softeners					
1.5% lipoic acid choline ester [30]	225	Multi-center, randomized, placebo-controlled, double-masked, dose-ranging study	Twice daily binocularly	Change in Binocular DNCVA From Baseline at Month 3	NCT04806503	2

Abbreviation: BDCVA—Best-distance corrected visual acuity, DCNVA—Distance corrected near visual acuity, DVA—Distance visual acuity, PBOHB—pilocarpine, brimonidine, oxymetazoline, hyaluronic acid and bromfenac.

**Table 2:** Overview of advantages and disadvantages of each treatment method for presbyopia.

Method	Advantages	Disadvantages
Spectacles	Easy access Very low risk of ophthalmic and systemic adverse events Proven for long-term use	Temporary effect Inconvenience
Contact lenses	Convenient for those who regularly use contact lens	Temporary effect Need daily care of contact lens High risk of ophthalmic adverse events
Pharmacologic	Easy to use No spectacles or contact lens needed No surgical risk	Temporary effect Risk of ophthalmic and systemic adverse events Required long-term application
Surgical	Can be permanent Low risk of systemic adverse events	No proven standard procedure

## Data Availability

Data sharing is not applicable to this article.
